# Structure-Function Discrepancy: Inhomogeneity and Delays in Synchronized Neural Networks

**DOI:** 10.1371/journal.pcbi.1003736

**Published:** 2014-07-31

**Authors:** Robert Ton, Gustavo Deco, Andreas Daffertshofer

**Affiliations:** 1 MOVE Research Institute Amsterdam, VU University, Amsterdam, The Netherlands; 2 Center for Brain and Cognition, Computational Neuroscience Group, Universitat Pompeu Fabra, Barcelona, Spain; 3 Institució Catalana de la Recerca i Estudis Avançats (ICREA), Universitat Pompeu Fabra, Barcelona, Spain; Brain and Spine Institute (ICM), France

## Abstract

The discrepancy between structural and functional connectivity in neural systems forms the challenge in understanding general brain functioning. To pinpoint a mapping between structure and function, we investigated the effects of (in)homogeneity in coupling structure and delays on synchronization behavior in networks of oscillatory neural masses by deriving the phase dynamics of these generic networks. For homogeneous delays, the structural coupling matrix is largely preserved in the coupling between phases, resulting in clustered stationary phase distributions. Accordingly, we found only a small number of synchronized groups in the network. Distributed delays, by contrast, introduce inhomogeneity in the phase coupling so that clustered stationary phase distributions no longer exist. The effect of distributed delays mimicked that of structural inhomogeneity. Hence, we argue that phase (de-)synchronization patterns caused by inhomogeneous coupling cannot be distinguished from those caused by distributed delays, at least not by the naked eye. The here-derived analytical expression for the effective coupling between phases as a function of structural coupling constitutes a direct relationship between structural and functional connectivity. Structural connectivity constrains synchronizability that may be modified by the delay distribution. This explains why structural and functional connectivity bear much resemblance albeit not a one-to-one correspondence. We illustrate this in the context of resting-state activity, using the anatomical connectivity structure reported by Hagmann and others.

## Author Summary

Separating the time scale of oscillations from that of the phase dynamics allowed for reducing a network of coupled neural mass models to a system of phase oscillators. We studied the dynamics of networks of phases and their synchronization characteristics as being seminal for functional neural networks. We put particular focus on effects of time delays in the coupling on the network dynamics and contrasted that to effects due to altered structural connectivity. Does neuroanatomical structure prescribe all the macroscopic activity patterns that we observe through electrophysiological brain recordings? We found that heterogeneity in structural coupling and distributed delays have equivalent effects on the shape of phase distributions, i.e., on functional connectivity. The contribution of changes in structural connectivity to network synchronization can therefore not readily be distinguished from that of distributed delays. Interestingly, the emergence of phase clusters in networks requires a subtle interplay between coupling and delays, which may form a window into disentangling structural effects from those induced by delay distributions. Therefore, when investigating neural network behavior, both structural connectivity and delay distribution should be addressed.

## Introduction

Much of the current focus in the empirical study of large-scale neuronal networks has been on their intrinsic activity and the degree to which the coherent patterns of this intrinsic activity reflect anatomy. The use of fMRI and diffusion spectrum imaging has allowed for a comprehensive evaluation of the structure-function map of resting-state networks (RSNs). In fMRI the spatial patterns of spontaneous changes in blood oxygenation level-dependent signals seem to reflect the generating neural architecture of RSNs. Despite the very slow changes of these signals, Biswal and co-workers [1] defined RSNs as networks of brain areas that exhibit *temporally coherent activity* in the absence of identifiable externally imposed or measurable events. More recently, RSNs penetrated the field of encephalography [2], [3]. For M/EEG, locally synchronized neural activity is considered to yield macroscopic oscillations that provide a basis for defining functional brain networks [4]. In most studies, structural connectivity is considered a good predictor of functional connectivity [5], [6]: Structural connectivity agrees with the anatomical connections between network nodes and functional connectivity covers the statistical relationship of nodal activity.

The predictive value of structure for function found support in recent modeling work using full brain systems with realistic anatomy, which demonstrated the structural dependency of functional network configurations [7]. There, functional connectivity has been estimated between all nodes over several hundred seconds of simulated time yielding the pattern of functional connectivity over this time window that largely reproduced the structural connectivity. At smaller time windows, however, shorter-living patterns of functional connectivity emerged that had not been predicted by anatomy. To understand this discrepancy we investigated effects of time delays vis-à-vis effects of structural inhomogeneity on synchronization patterns of neuronal networks.

Delays are inherent in neuronal networks due to finite conduction velocities [8] and synaptic transmission [9]. Ignoring delays may be a valid starting point for mathematical analysis but when doing so one runs the risk of loosing biological plausibility. However, incorporating delays in oscillatory networks does come with immense challenges. Already for low-dimensional oscillatory systems (or for high-dimensional ones with strong symmetry) the presence of delays is known to change the dynamical repertoire significantly [10], [11]. Yeung and Strogatz showed for very large networks how time delays can alter synchronization properties, even if the structure is isotropic and homogeneous [12]; see also [13], [14]. Numerical assessments revealed similar results for biologically motivated and hence more inhomogeneous connectivities. Delays seem to be crucial in establishing the spatio-temporally organized fluctuations typically observed in resting state brain recordings [15]–[17]. In the present study we sought to tackle this issue and separated the effect of time delays from that of inhomogeneous connectivity by studying networks consisting of distinct neural masses. Neural mass models offer a low-dimensional description of the dynamics of a large neuronal population and exist in a variety of forms [18]. We chose for Freeman's seminal model [19]–[21], since it covers the dynamics of mean membrane potential changes that relate closely to encephalographic signals. A network of such entities may constitute RSNs if we regard the neural masses to be representative of individual brain areas.

Throughout the paper we describe functional connectivity by means of phase synchronization whose dynamics can be estimated in voltage-based and firing-rate models using a combination of *rotating wave* and *slowly varying amplitude* approximations, or in brief *averaging,* see [22], [23]. In the *Methods* section this combination of approximations is briefly summarized for Freeman neural mass models in the oscillatory regime. Central outcome measure is thus the phase dynamics of the individual nodes in the network or, to be more precise, the density of the nodes' phases as a function of time, often also referred as time-dependent *population distributions*. We note that we applied this approach before to instantaneously coupled Wilson-Cowan firing rate models [24] (see also [25]) but, as said, we here chose for the Freeman model for an easier comparison with M/EEG studies. For coupled Freeman models we could analytically determine the corresponding stationary distributions even in the presence of delays and inhomogeneous coupling between neural masses. We could not only prove the existence of these solutions, but we were also able to determine the loss of stability of the desynchronized state as soon as the overall coupling strength exceeded a critical value. More complicated scenarios including biological plausible anatomical adjacencies were treated numerically to illustrate the non-trivial relationship between structural and functional connectivity.

## Results

We considered a set of 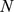 coupled neural masses whose mean membrane potentials 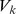 follow the dynamics 
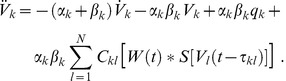
(1)


**Figure 1:**
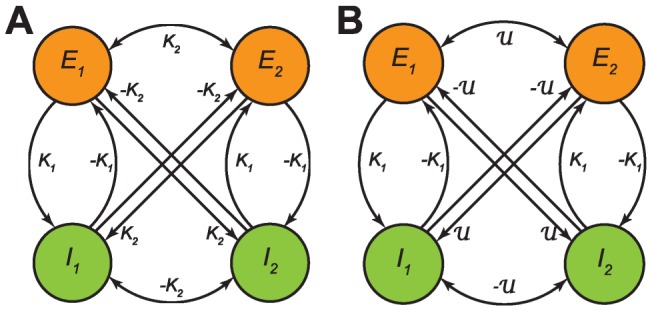
Structural connectivity in the case of two coupled pairs of excitatory/inhibitory neural masses. **A**: Homogeneous coupling with coupling matrix 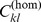. **B**: Inhomogenous coupling using the coupling matrix 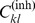. In both cases excitatory/inhibitory-pairs have a symmetric ‘internal’ coupling with strength 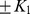 but the coupling between these pairs differs. In A the between-pair coupling is homogeneous at strength 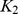 whilst in B the between-pair coupling may differ across pairs; i.e. it is randomly chosen from a certain distribution 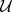 with the constraint that inhibitory units map (on average) with negative and excitatory with positive coupling strength. See text for more details and Figs 7 and 8A for the more complicated coupling schemes employed below.

In this expression 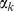 and 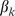 represent mean rise and decay times of neural responses in population 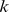, 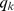 stands for an external input, and 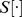 denotes a sigmoidal activity function covering the effects of pulse-coupled neurons in populations 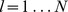 [19], [26]. Corresponding mean activities 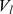 arrive at population 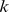 after yet arbitrary delays 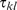. The structural connectivity matrix 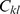 served to introduce both excitatory and inhibitory connections in the thus asymmetric coupling; see Fig 1A. We first considered the case in which a large degree of homogeneity was present in 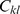 to define a ‘baseline’. Subsequently we introduced inhomogeneity to mimic, e.g., the sparse connectivity presumably underlying RSNs. Two seminal coupling schemes are sketched in Fig 1. Excitatory and inhibitory populations were always properly balanced to stabilize oscillatory behavior [27], [28]. This translates to the condition that at least one pair of the eigenvalues of the linearized system around the fixed points 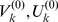 must be imaginary with positive real part. From the *Methods* section it follows that the considered coupling schemes did satisfy this condition.

For the convolution we chose an exponentially decaying kernel 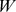


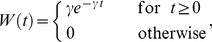
(2)to represent the dynamics at the synaptic junction. In the particular case of infinitesimal memory, i.e. for 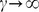, we found the corresponding phase dynamics by transforming the neural mass dynamics to polar coordinates around an unstable focus, i.e. 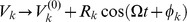; 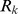 denotes the amplitude of oscillation and 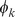 its phase corresponding to the central frequency 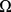. Subsequently, we averaged the dynamics over one period 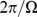. We assumed that the characteristic times of amplitude and phase dynamics are significantly larger than the period 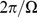, i.e. amplitude and phase dynamics are slow compared to the oscillation. We also assumed the time delays to be of the same order of magnitude or smaller than the period. As a result the delays 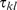 reduced to mere phase shifts 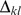 and the phase dynamics became 
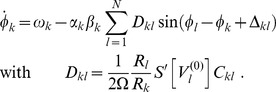
(3)


**Figure 2:**
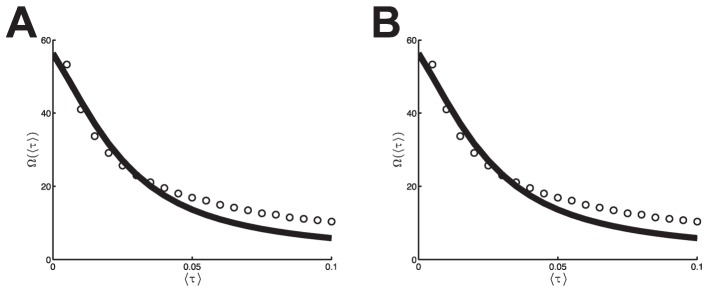
Frequency and period as a function of delay. **A**: Oscillation frequency 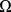. **B**: Period 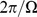. The analytical estimate under small-delay approximation (see *Methods*) is displayed as a solid line; results of numerical simulations of the dynamics (1) as dots (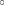). Simulations were performed for homogeneous coupling 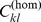 with within-pair coupling 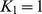, between-pair coupling strength 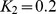, and distributed delays 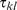; see text for details. The presence of between-pair coupling 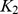 and even heterogeneity in coupling, 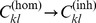, did not yield qualitatively different results.

In the dynamics (3) the frequencies obeyed the form 
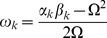
 and the phase shifts read 
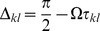
. As said, delays could be transformed to phase shifts due to a time scale separation in the system, i.e. the phase dynamics was set sufficiently slow compared to the oscillation and the delays 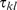 were up to the same order of magnitude as one period of oscillation (cf. Fig 2).

At first glance the dynamics (3) seemed to largely resemble the Kuramoto network of phase oscillators that is well known for its bifurcation scheme from desynchronized to synchronized states. The latter, i.e. the fully synchronized state, would imply large-scale — if not whole-brain — synchronization, which, apart for very pathological cases, is not observed experimentally. Nonetheless we considered linking our study to Kuramoto's profound work to be very insightful, as understanding the Kuramoto network is essential for understanding synchronizability in our more general framework. A closer look revealed that, although similar, (3) differs from the Kuramoto network in two important aspects. First, in the phase dynamics the coupling is given by 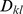, which in general is not entirely homogeneous as in Kuramoto's case. This expression for 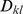 agrees with the previously derived phase dynamics of coupled Wilson-Cowan oscillators in that the amplitude relation 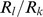 between nodes 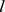 and 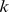 affects the corresponding (relative) phase [24]. Second, the finite delay 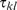 yields a non-trivial phase shift 
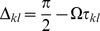
. This phase shift might alter the spectrum of phase synchronization entirely; see, e.g., [29], [30] for the case.

Instead of studying the mere collection of phases 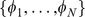 we investigated the dynamics of their probability density 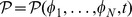, because it forms a proper measure of synchronization. To explain: Synchronization around a certain phase value 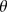 manifests itself as a peak in the probability density around that value, i.e. a phase cluster around 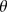. For our set of Freeman neural masses we found that the phase distribution follows the dynamics 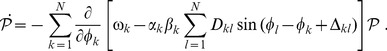
(4)


This is the continuity equation, which equals the zero-noise limit of the corresponding Fokker-Planck equation when presuming ergodicity and very large networks (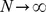). Despite the presence of delays 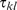 we here succeeded to specify stationary solutions 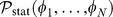. Crucial in finding these solutions was the fact that they can always be written as a mere sum of distinct clustered states, i.e. they always obey the form 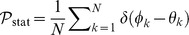, because the network has a countable number of nodes. Put differently, the phase distribution contains clusters centered around 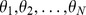. In general, the real number of clusters is given by the number 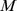 of distinguishable centroids 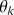; we always consider 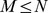. The number 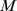 strongly depends on the distribution of delays and/or inhomogeneity of the coupling matrix 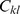 or strictly speaking of the effective coupling 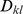.

In order to illustrate stationary solutions of (4) we first specified the coupling matrix 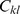 to be either homogeneous or inhomogeneous. In both cases we labeled excitatory and inhibitory populations by 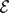 and 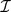, respectively, and defined the corresponding sets 
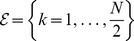
 and 
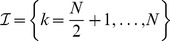
. As mentioned above we guaranteed throughout analysis that all excitatory populations had an inhibitory counterpart generating and stabilizing oscillatory activity at around the central frequency 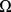 [27], [28]. For the sake of simplicity we ignored self-coupling of neuronal populations. The stationary phase distribution could thus be written as 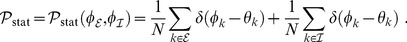
(5)


### Homogeneous coupling

In the homogeneous case we employed the coupling scheme sketched in Fig 1A. Excitatory and inhibitory populations were fully connected (apart from self-coupling) with coupling values 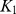 and 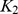 discriminating within-pair and between-pair coupling. For the numerical assessment we always fixed the within-pair coupling to 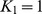. In more detail, we chose the overall homogeneous coupling matrix as 
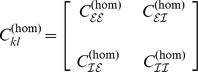
with sub-population connectivities 
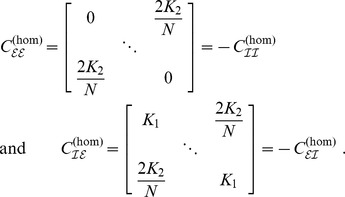



**Figure 3:**
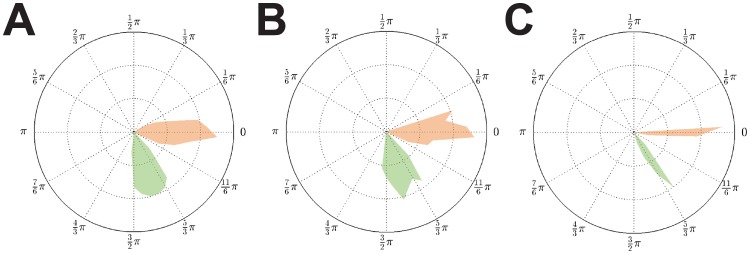
Phase distributions for strong coupling (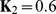) and equal delays. **A**: Vanishing delay (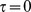). **B**: Fixed finite delay 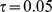. **C**: Fixed finite delay 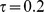. In all cases synchronized solutions 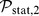 can be seen and appeared to be the only stable solution in the case of strong coupling 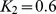. Phase distributions were obtained from simulations of the original system (1), where phases were extracted using the Hilbert transforms of 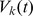; see *Methods* section. In this and all subsequent figures excitatory phases 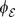 are depicted in orange and inhibitory phases 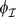 in green. Radial axes are normalized.

In the absence of delays, i.e. for 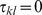 (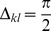) and sufficiently strong coupling 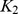 we found robust distributions with 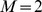 phase clusters, one containing all the excitatory populations and one all the inhibitory ones: 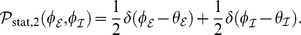



An example of this solution is illustrated in Fig 3A; the remaining panels in that figure refer to cases of non-vanishing delay that will be summarized below. We note that due to symmetry the homogeneous case with 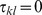 can be readily transformed, proving its resemblance with the Kuramoto network [31]. The somewhat lengthy analytic derivations are given in the *Methods* section.

Next to the homogeneously synchronized state 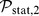 we found a solution with 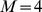 phase clusters given by 
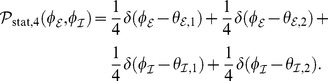



**Figure 4:**
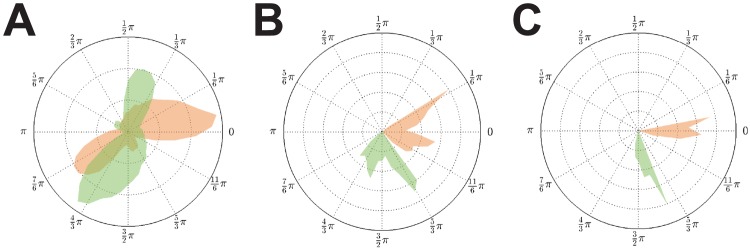
Phase distributions for weak coupling (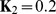) and equal delays. **A**: Vanishing delay (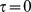). **B**: Fixed finite delay 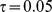. **C**: Fixed finite delay 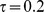. As for strong coupling we found synchronized solutions 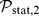 in the weak coupling case 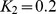. In addition solutions 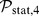 were also present. The number of clusters did depend on the delay value 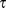. That is, altering the delay from 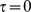 to 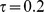 caused a switch in stability between these two solutions. Since for 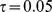 the two clusters within an 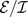-group only showed a small phase difference, we conjecture that 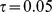 is close to the critical value of this bifurcation parameter.

Again we refer to the *Methods* for the analytical treatment with respect to the existence of this stationary solution; see Figs 3A and 4A for the corresponding numerical assessments. The specific form of the dynamics (3) and (4) already suggested that 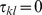 is just a special case of 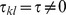. We therefore expected the existence of the solutions above not to be affected by introducing homogeneous, finite delays 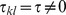, even if this appeared somewhat counterintuitive. In fact, numerics confirmed this expectation as displayed in Figs 3B, 3C, 4B and 4C.

**Figure 5:**
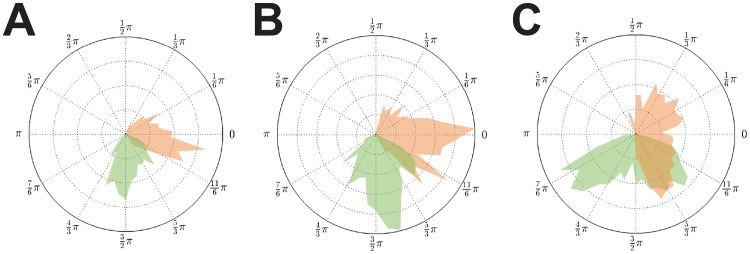
Phase distributions for different coupling strengths and distributed delays. **A**: 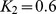, 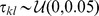. **B**: 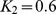, 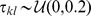. **C**: 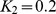, 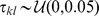. The effect of a delay distribution and consequently the presence of 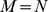 centroid phase values 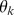 manifested itself as a widening of the phase distribution compared to the constant delay cases in Fig 4. Narrow delay distributions with 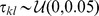, i.e. the 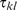 were randomly drawn from a uniform distribution over the interval 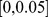, yielded comparably narrow phase distributions located around two narrow peaks (peaks representing the 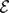
- and 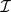-populations; (A)). Increasing the width of the delay distribution to 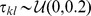 (B) had a very similar effect as lowering the coupling strength 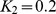 (C): in both cases the phase distribution widened substantially.

The introduction of distributed delays 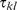 instead of a single 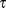 changed results profoundly. To exemplify this, we distributed delays 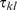 by drawing them at random from a uniform distribution over a certain interval. Recall that according to the transformation from (1) to (3), a distribution of delays 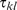 generally implies an equivalent distribution of phase shifts 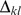. If 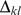 differed for all populations 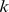 and 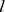, the stationary solution 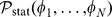 of the continuity equation (4) required the presence of many distinct phase clusters. We could prove the existence of that set and, although the generic solution appeared similar to the homogeneous delay case 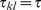, it did contain 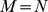 centroid values 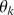 instead of the small number 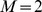 or 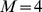 shown above. We depict examples of phase distributions for several parameter settings in Fig 5. Interestingly, the heterogeneity in 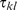, or equivalently in 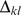, agreed with weakening the between-population coupling 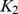 in that both cause a profound widening of the phase distributions; compare Fig 5A with 5C. That is, for a network with homogeneous structural connectivity, it is not the presence of delays per se that hinders synchronization but rather the distribution of delays (or the lack of coupling strength).

### Inhomogeneous coupling

According to (3) both distributed phase shifts (or delays) and heterogeneous coupling may in principle result in inhomogeneity of phase coupling. In other words, distributed delays 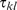 and structural heterogeneity may yield inhomogeneity in functional connectivity. We therefore expected a heterogeneous coupling matrix with homogeneous delays 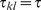 to be accompanied by desynchronization equivalent to the case of homogeneous coupling and distributed delays. To verify this, we used the inhomogeneous coupling sketched in Fig 1B, which can be given more formally as 
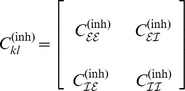
with sub-population connectivities 
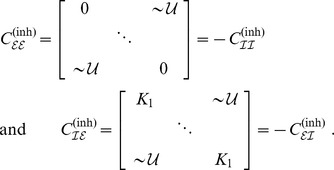
where 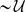 abbreviates 
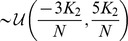
 denoting that the off-(sub)diagonal entries were randomly drawn from a uniform distribution centered around 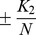.

**Figure 6:**
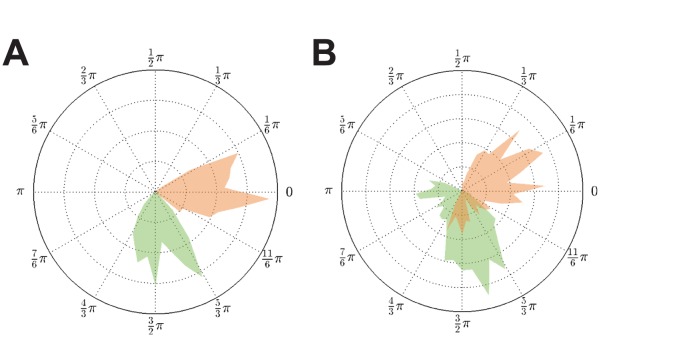
Phase distributions in the presence of inhomogeneous coupling 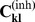. **A**: Strong coupling (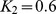). **B**: Weak coupling (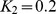). As predicted by the phase derivation (9), inhomogeneity in the coupling matrix resulted in similar behavior as a distribution in delays; compare Figs 5A and 5C with panels Fig. 6A and 6B respectively. For reasonably weak coupling we found a widening of the phase distribution equivalent to the case 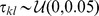 with homogeneous coupling. Again, an increase in coupling strength resulted in a concentration of 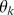.

The numerical simulations depicted in Fig 6 confirmed our hypothesis. For 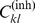 and 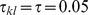 we observed a widening of the phase distribution similar to that shown in Figs 5B and 5C where coupling was established by 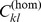 but with delay distributions 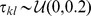 and 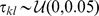 respectively. Increasing coupling strength reduced the width of the phase distribution comparable to the switch from Fig 5C to 5A. Functional connectivity thus seems to result from an interplay between structural connectivity 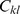 and delay structure 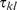. Therefore both should be taken into account when studying functional connectivity in neuronal networks.

### Anatomical coupling

**Figure 7:**
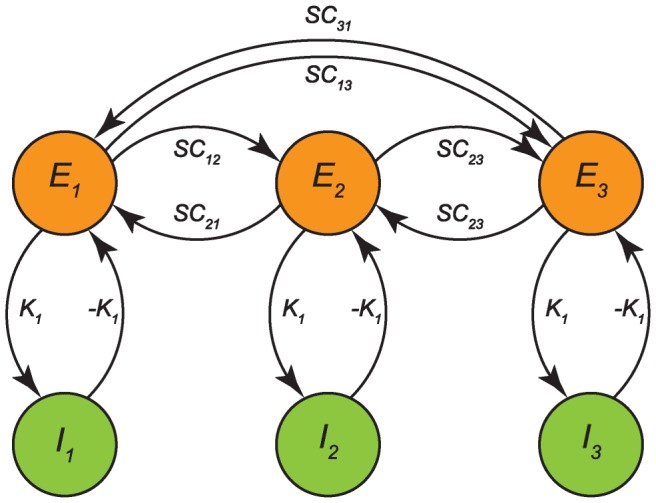
Neural mass coupling scheme for anatomical coupling 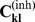. For 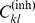 the SC matrix determines the coupling between excitatory units only. The SC matrix is the neuroanatomical coupling matrix; here three coupled excitatory/inhibitory pairs are shown. The other connections between excitatory and inhibitory masses serve to establish oscillatory behavior, resulting in a coupling matrix that is relatively sparse compared to 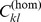 and 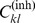.

The coupling matrices 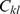 considered so far were admittedly quite academic. However, these seminal examples did provide important insights that | as we will show here | generalize to more complicated and biologically plausible cases. We performed simulations using the coupling scheme displayed in Fig 7. The matrix 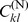 had the same structure as 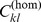 but now the blocks were given by 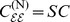, 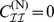, and 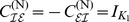. The acronym SC stands for ‘structural connectivity’ that here refers to a neuroanatomical connection matrix as can be derived using DTI/DSI imaging [32], [33] and 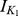 is the identity matrix with 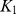 along the diagonal. To be precise, we used a binary form of the Hagmann connection matrix; see [24] for specifics of pre-processing.

**Figure 8:**
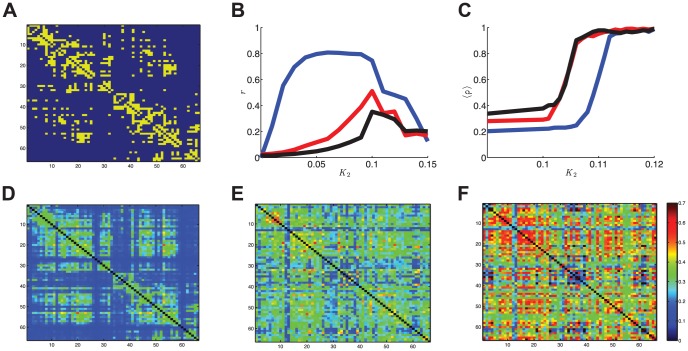
Anatomical connectivity matrix and resulting functional connectivities as a function of delay distribution. **A**: Sparse neuroanatomical coupling matrix 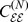 serving as structural connectivity (connections are given in yellow). **B**: Structure-function correlation 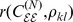. **C**: Overall synchronizability 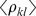. Between-pair coupling strength 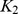 appears on the horizontal axis; while blue, red, and black lines correspond to 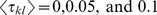 respectively. Note that an increase in delay distribution width increased synchronizability due to increased 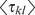, but the accompanying higher 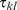 variance decreased structure-function correspondence as predicted by (3). **D-F**: Corresponding spatial synchronization matrices 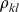 for delay distributions 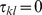, 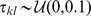 and 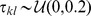 respectively (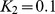). Color coding is displayed in the most right panel; only excitatory nodes are shown.

In line with earlier studies we quantified functional connectivity in terms of *phase uniformity* or *phase locking value* of the pair-wise relative phases, i.e. 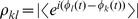. Using this synchronization measure, simulation results can be best summarized in the form of functional connectivity matrices constructed from 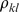 values for all available pairs. The effects of delay structure 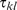 on these functional connectivity matrices are depicted in Figs 8D-F with the underlying structural connectivity given in Fig 8A. The functional connectivity matrix appeared rather sensitive for parameter values, as increasing coupling strength from 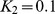, which was the value used in Fig 8D-F, up to 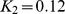 resulted in a fully synchronized network as can be seen in Fig 8C. The sudden synchronization is reminiscent of the phase transition towards the fully synchronized state at the critical coupling strength 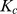 in the Kuramoto network.

In a nutshell, from (4) we could deduce the mechanism responsible for the general finding that structural and functional connectivity are positively correlated [5], [6]; see also Fig 8B. Our results clearly show that delay distribution affects both the spatial distribution of functional connectivity (Figs 8B; 8D-F) and the overall level of synchronization in the network (Fig 8C). The increase of overall synchronization is caused by a decreased phase shift 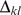 by which the phase dynamics (3) converges towards the Kuramoto model, i.e. the delay induces a change in stability of the (partially) synchronized state.

## Discussion

We investigated the effect of time delays in the coupling between neural mass dynamics, where we consider an oscillatory regime, established by creating pairs of excitatory/inhibitory neural masses. Although we employed a specific neural mass model, we do consider our results generic because the mappings 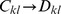 and 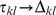 are largely independent of the generating nodal dynamics, presuming that the time scales in the system are sufficiently separated; cf. *Methods* *.*

By using this oscillatory dynamics to describe activity in certain brain areas, our approach links directly to the ongoing dispute about changes of functional connectivity in resting state networks (RSNs). There is growing evidence from experimental research that spontaneous brain activity during rest is highly structured into characteristic RSN patterns [1], [3], [34], [35]. These activity patterns seem not to be the result of structural connectivity alone [5], [36], but to reflect a non-trivial interplay between the neuroanatomical structure and dynamics [37]. The distribution of time delays involved in this dynamics may have an important role in shaping patterns of activity per se and neuronal synchronization in particular [15]–[17], [38].

Key to our analysis was the reduction of a neural mass network to a system of phase oscillators summarized in (3). Several previous studies struggled with computational complexity when trying to unravel effects of delays vis-à-vis coupling on network dynamics [15]–[17], [38]. By contrast, our analytic reduction ‘readily’ allowed for disentangling the contributions of both structural connectivity 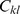 and delays 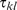 to the phase dynamics (3). Delays 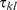 entered the phase dynamics as phase shifts 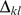, given a proper time scale separation of oscillatory and phase dynamics. Furthermore, we found that heterogeneity in delays yields effects equivalent to those of heterogeneity in structural connectivitiy. That is, connectivity and delay effects cannot be easily distinguished when solely looking at functional connectivity patterns.

The decrease of 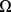 as a function of delay, as depicted in Fig 2, agreed with the analytical findings of [13] as well as with our small-delay approximations outlined in the *Methods*. However, when further comparing the current results with the literature, one has to realize that some fundamental differences exist between general phase oscillator networks and our dynamics (3). One of those differences is the finite dimensionality of the system (3). We assumed every excitatory/inhibitory neural mass pair to represent a single brain area, by which the dimension of the system under study may be fairly low. On the contrary, most analytical work on phase oscillator networks considered the limit 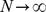 [14], [39]–[41] rendering one-to-one comparisons all but trivial. This can already be appreciated by the rather dramatic finite-size effects in Kuramoto networks [42]–[45]. Moreover, our structural connectivity 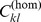 is rather atypical due to its strong 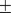-asymmetry. Usually, the connectivity structure in similar phase oscillator networks comprises either fully homogeneous coupling or the entries 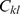 are distributed according to some unimodal distribution [42], [46]. An exception is [47], who investigated repulsive coupling, which is similar to the excitatory/inhibitory connections in 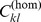. That study reported the presence of two anti-phase clusters reminiscent of the separate 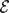/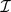-groups observed here.

The time scale separation in (3) and the resulting simplification of delays 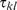 as phase shifts 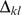 also hinders direct comparison with studies on delays in the Kuramoto network. This may indeed explain our seemingly contradicting results. For example, we did not observe the emergence of multi-stability mediated by specific (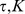)-combinations as reported in [12], [48]. In those studies, regions in the (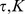) phase plane were found in which synchronization was entirely absent. This is clearly not the case in our study. We did find synchronized solutions irrespective of delays. This is not trivial, because for 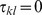 the phase dynamics equation (3) may be reduced to a cosine-variant of the traditional Kuramoto model, which is known for its inability to display synchronized behavior [49]. By exploiting the pairing of the 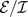-groups and the 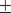-asymmetry in 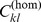 we could map our averaged neural mass network to a conventional, fully homogeneous Kuramoto network via a mere transformation of variables. Hence our system can display synchronized behavior even for vanishing 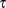 values. This is consistent with [50] who did not find any qualitative effects of a phase shift 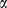 on the stability of the Kuramoto network.

Apart from choosing random values as entries of the 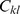-matrix, inhomogeneous coupling might also stem from creating distinctively different sub-populations in the network. It can then be studied by modifying within- versus between-network interactions. Particularly interesting in this respect is the occurrence of clustering in the network. When delays are not incorporated one needs either structural inhomogeneity [51] or higher-order Fourier harmonics, in combination with an appropriate phase shift [30], to achieve clustering. The phenomenon of clustering is important in the light of the study of RSNs, i.e. the strong spatiotemporal organization observed in brain activity during resting state conditions [1], [3], [34], [35].

We briefly consider a simple, low-dimensional example of three isolated excitatory nodes. We define a cluster as a number of (excitatory) masses attaining the same centroid phase value 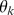. By denoting the excitatory and inhibitory centroid values as 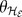 and 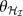, respectively and assuming 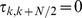, from the phase derivation (3) and its corresponding continuity equation (4) one finds constraining equations 
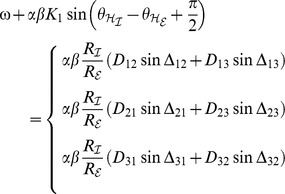
for 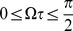. In fact, these forms already hint at the interference between coupling and delays and its effect on synchronization structure. First, all terms on the right-hand side must have equal magnitude requiring specific combinations of 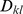 and 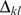. However, both 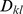 and 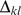 are constrained by biology: 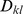 by the neuroanatomical coupling 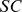 as part of 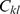; and 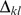 by the spatial structure of the brain, as delays are proportional to distance between masses 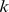 and 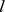 due to finite conduction velocity. Second, because the left-hand side does not vanish, 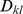 must have some lower bound. If 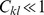, then 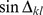 cannot compensate this and the equality cannot be satisfied. Because 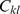 determines 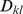, there must be some minimal coupling strength between nodes for synchronization to emerge. This explains the positive correlation between structural and functional connectivity, see, e.g., [6], [17], [38]. It also shows the intricate interplay between structure and delays in establishing synchronization structure.

Interestingly, 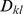 may be regarded as the effective coupling matrix that is typically encountered in dynamic causal modeling approaches [52]. The fact that 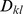 is directly determined by 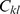 also explains the finding that models using the structural connectivity as a prior do show more evidence than models using other priors [53]. That is, models that have structural connectivity as a starting point, perform better in terms of data explanation. The sparsity of 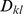 induced by 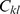 may yield coexisting synchronized and desynchronized groups within the network, which are often labelled chimera states in the study of phase oscillator systems. It has been found that they crucially depend on the combination of coupling strengths and phase shifts [45], [54], [55] (or delays [56]), confirming that there has to be a specific matching of coupling and delays for synchronization to occur.

Against the background of the aforementioned 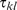-dependence of functional connectivity and the functional-structural connectivity correlation for a biological plausible network, we numerically investigated this by performing simulations of (1) with structural connectivity given by the anatomical connectivity matrix 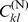 reported by Hagmann and co-workers [32]. Functional connectivity was quantified as pair-wise phase uniformity, i.e. the phase locking value. Our numerical assessment is summarized in Fig 8. It clearly revealed off-diagonal patches with synchronization between nodes that are not coupled (contradicting what has been sketched above). The topology of the Hagmann et al. network shares similarities with the Watts and Strogatz' small-world network [57], i.e. both have a relatively large clustering coefficient with a small average path length. This kind of topology is often believed to be generic in biological neural networks like our brain [5] and enhances synchronizability compared to random networks [57]–[59]. The presence of sparsely connected clusters establishes synchronization between nodes that are only indirectly coupled via their clusters. This causes ‘blurring’ of the structural connectivity matrix: The functional connectivity matrix is less sparse than the structural one [60]. Although this ‘blurring’ is similar to the effects attributed to volume conduction [61], in this case it is solely due to network topology.

Next to such clustering phenomena, we can make even more general predictions about the effect of delays in this network. Structural and functional connectivity are most prominently correlated for homogeneous delays, since 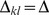 yields an interaction term in (3) that is merely a scaled version of 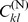. Hence, the resulting spatial synchronization distribution largely resembles 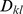 and thus 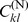, presuming that the overall coupling strength is not excessively large. This effect can be seen in Fig 8B, where we depicted the Pearson correlation coefficient between the lower triangular parts of 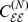 and the functional connectivity matrix 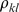. Increasing the width of the delay distribution results in a decrease in structure-function correspondence. The positive correlations are consistent with the finding that the pattern of resting state activity is spanned by the eigenmodes of the underlying connectivity matrix in [62]. This is not as trivial as it may seem because the node dynamics in this study were noise-driven fluctuations around a stable fixed point and therefore entirely different from the self-sustained oscillations considered in the current study.

Widening the delay distribution also had another effect: It increased its average value and consequently the mean phase shift 
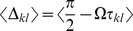
 tended to vanish. Therefore, the interaction term 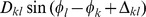 became more similar to an ‘ordinary’ sine-term, which is known for its capacity to enhance synchronizability [63]. We illustrate this effect in Fig 8C, where overall synchrony 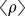 is shown as a function of coupling strength 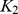 for different average delay-values 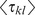. A similar phenomenon has been reported for a system of coupled Hindmarsh-Rose neurons, where a stable synchronized region appears to exist despite the presence of a (constant) delay [64].

Throughout this study we assumed the amplitudes to be constant. The relation between the envelope dynamics of M/EEG and fMRI-BOLD signals [3] suggests that considering the temporal change of the amplitude may be very important for unravelling the spatio-temporal structure of resting state brain activity. Given that we focused on phase synchronization together with the slow time scale on which the BOLD dynamics evolve (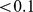 Hz, [1]), we believe that the assumption of constant amplitude is justified here. Investigating this assumption in depth, however, is beyond the scope of the current study.

We summarize that the dynamics of a system of coupled Freeman neural masses (1) can be captured by the averaged phase dynamics (3), in which the role of the structural connectivity 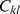 and delay distribution 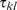 become explicit. By this, one can identify the relative contributions of structure and delay to phase synchronization, i.e. to the functional connectivity of the neural network. Heterogeneity in structural coupling and distributed delays have equivalent effects on the observed phase distributions. Overall, this supports the notion that structure and delay are both crucial determinants of network behavior and should therefore be taken into account in unison whenever modeling realistic neural networks [37]. Our examples on clustering detailed how the intricate interplay between coupling and delays determines the form and spatial distribution of clustering in these networks. Pinpointing the explicit contributions of 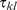 and 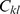 in the phase dynamics (3) enabled us to understand their roles in establishing synchronization structure and why functional and structural connectivity are so closely correlated. This implies that the observed temporal changes in synchronization structure in resting state and task conditions can be modulated through either 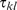 or amplitudes 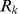.

## Methods

To analyze the gross membrane voltage of a neural mass we first wrote its dynamics (1) as a two-dimensional system using the auxiliary variable 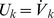. For the sake of simplicity we further rewrote the convolution integral in (1) by means of 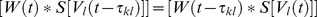. Then the dynamics (1) reads 
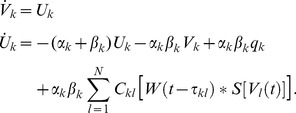
(6)


In the following we discuss this dynamics in its oscillatory regime after transforming the system into polar coordinates to derive the corresponding phase dynamics. That transform, however, was not applied to the original state variables 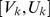 but to the deviations 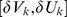 around the unstable fixed points 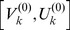, i.e. we transformed 
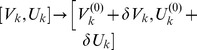
. Furthermore, we expanded 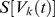 around 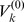 in the sense of Taylor and obtained 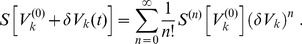
(7)


With this expansion the dynamics of the deviations 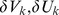 could be approximated as 
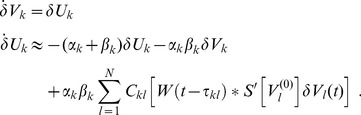
(8)


A closer look at this linear system revealed that the fixed points 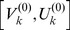 were indeed unstable nodes, provided that a proper balance between excitatory and inhibitory masses was present. That is, the eigenvalues of the linear system (8) were complex-valued with positive real-parts — explicit expressions for the eigenvalues (as a function of delay) can be found below.

Next, we transformed system (8) into polar coordinates by means of 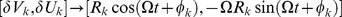 so that the phases could be cast into the generic dynamics 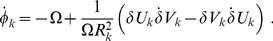
(9)


Importantly, in the current case we could assume that the phase dynamics (9) in general contains two (or more) distinct time scales: the rate of change given by the oscillation defined via the frequency 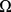 and the time scale(s) of the amplitudes 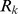 and the phases 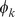; the latter are much slower than 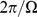 and can hence be separated. More formally we used 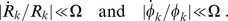



In first order approximation we could thus consider 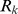 and 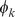 to be constant during one period of oscillation, 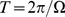. This approach is referred to as a combination of a *rotating wave approximation* and a *slowly varying amplitude approximation* [65]. It enabled us to average the dynamics over the interval 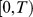; see also [23]. As will be shown in the following, this averaging procedure decoupled amplitude and phase dynamics, which ultimately resulted in the dynamics (3). Below we will provide a more formal discussion regarding these approximations.

### Averaging—towards the phase oscillator model

To average the dynamics (9) we defined the averaging operator as 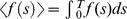. We first substituted (8) into (9) and used 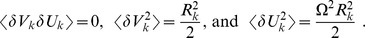



The convolution integral on the right-hand side of the second equation of (8) required more attention. Recall the definition of 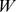 in (2) and the definition of the convolution operator, with which one can write 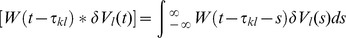


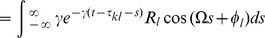


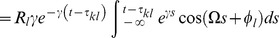


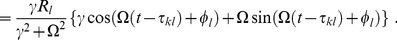
(10)


When multiplied by 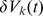, this yielded the two averaged trigonometric expressions 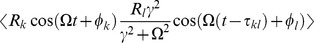


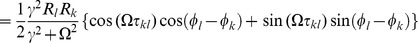
and 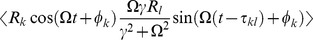


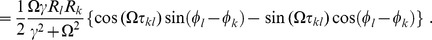



After substituting this in (9) we obtained the phase dynamics 
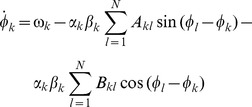
(11)where we defined the constants 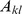 and 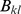 as 
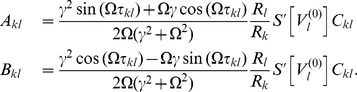



By this procedure we omitted all fast oscillating terms as they averaged out (cf. *rotating wave approximation*).

As mentioned in the *Results* section, we focused on the case in which our convolution kernel 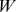 did not contain any memory. That is, we considered the limit 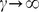. In this limit only the first terms in the numerators of 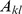 and 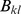 remained non-zero and we could cast (11) in the form (3) using 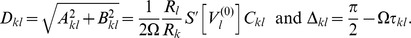



Note that in this form, the delays 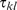 only appeared in the phase shift 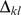. Last but not least we simplified expression (3) by exploiting the homogeneity of 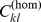 to explicitly formulate the 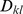 matrix multiplication. In particular for equal delays, i.e. for 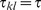, this led to a greatly simplified form of (4) that we summarized in the *Results* section and will be discussed in more detailed below.

### Fixed points and amplitudes

The neural masses do not oscillate around the origin but around the fixed points 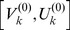, which have a direct influence on the coupling terms 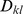 through the term 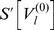. Delay values 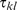 do not influence the positions of the fixed points 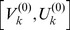 because by definition 
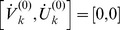
. Hence, 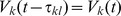 holds, presuming the fixed points exist. Therefore we were free to choose 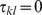, such that under the limit 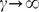 the coupling term in (6) reduced to 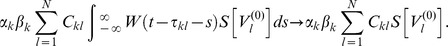



After inserting the form of 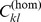 we explicitly found 
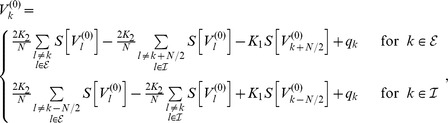
which implied 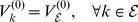 and 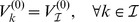. That is, in the case of the homogeneous coupling the fixed points of the excitatory masses are equal and the same holds for the inhibitory masses.

The coupling 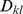 also depended on the amplitudes 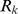 of the neural masses — see also [24]. Accounting for the high degree of homogeneity in the system, we assumed the amplitudes to be equal for equal types of neural masses, i.e. 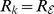, 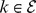 and 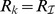, 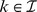. Furthermore we randomized the parameters 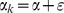, 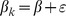 by introducing 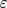 as a mean-centered random variable. Whenever appropriate we chose 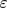 sufficiently small to restrict discussion to the mean values 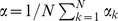 and 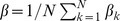.

### Homogeneous coupling — existence of solutions

Since the phase oscillator system (3) can be cast in Kuramoto form, fully synchronized solutions may be stable despite the presence of equal delays 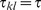. But how about solutions other than the fully synchronized ones? In what follows we discuss existence and form of partially synchronized solutions of (3) for general delays 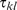. We concentrated on homogeneous coupling and varied the distribution of 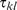. In the homogeneous case we found the dynamics of the 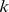-th node's phase distribution 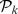 to be 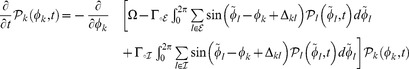
(12)where the subscript 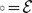 when 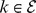 and 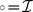 when 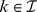. Note that equation (12) may differ for every 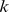. Homogeneity of 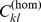 enable us to express 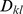 explicitly and to define the following constants 
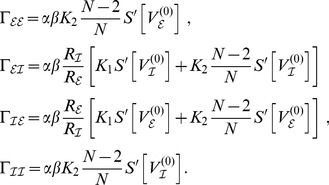
(13)


Sufficient for the existence of a stationary solution is the case in which the drift coefficient in the dynamics of the probability density vanishes, here the bracketed term on the right-hand side of (12). From the dynamics (12) it readily follows that the phase distribution obeys the form (5), i.e. 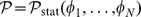, containing 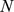 different centroid phase values 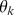.

#### Equal delays


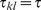
 **.** As said, for homogeneous coupling the sums in (12) could be evaluated in the form of the constants in (13). Furthermore in the case of equal delays 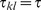 (i.e. 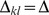) we only had to account for two distinct populations each with just a single type of density dynamics, by which the system (12) of 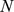 equations could be reduced to merely two distinct ones: 
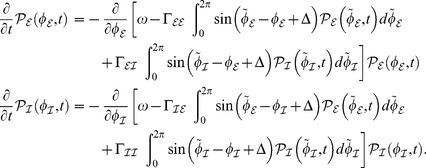
(14)


To show the existence of 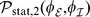 consisting of two phase clusters, one for the excitatory units and one for the inhibitory units, i.e. 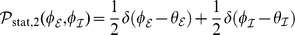
we followed a constructive approach and substituted 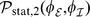 into (14). Vanishing of the drift coefficient required 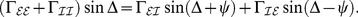
(15)when abbreviating 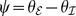. Using 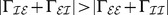, which followed from (13) irrespective of the value of 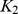, we could conclude that a solution 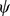 satisfying (15) exists. Note that only 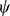 appeared in (15) and not the centroid values 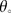, which allows for the mapping to the conventional Kuramoto model as will be discussed below.

We could readily generalize this line of reasoning to an arbitrary number of clusters after defining the stationary phase probability density containing 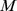 clusters as 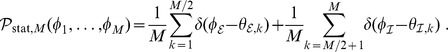



We defined generalized phase differences 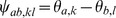 where 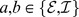 and 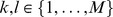, by which we obtained the set of constraining equations as 
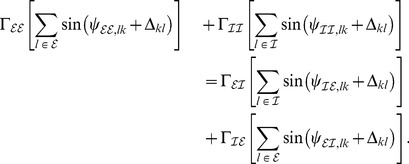
(16)


In contrast to (15) we here considered general delays 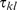, but note that the form of (16) did not change for 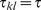. As the values of the constants 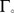 still fulfilled the inequality 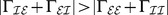 we again could conclude that this system can be solved. Thus, even for equal delays 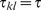 (or 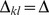) the stationary phase distribution may contain any number of clusters 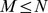. Of course, this existence proof does not imply that all these solutions will be found in reality since we have not yet addressed their stability; cf. Figs 4A and 3A. Note that apart from the fully synchronized state, we here restricted our stability analysis to numerical evaluations.

The distributed delay case (16) does not require 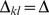. Hence, for general 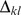 the stationary distribution 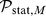 will contain 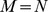 centroid values 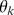 with 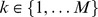.

#### Stability for 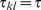 — a fling with the Kuramoto network

Before proving the existence of distinct solutions in the case of arbitrary delays 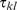, we first briefly discuss the case of constant delay because it readily links the model (3) to the Kuramoto network of coupled phase oscillators [63]. For 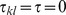, the interaction term in (3) reduces to a mere cosine term because of 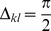. We note that in Kuramoto's traditional form this cosine term leads to a system that is unable to show synchronized solutions [49]. Our numerical simulations, however, revealed synchronized solutions; see, e.g., Figs 3 and 4. In what follows we show that due to the fact that the centroid phase values 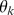 are irrelevant one can distill the traditional Kuramoto model.

It is the coupling matrix 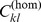 that made the difference with Kuramoto's network as we assumed a (balanced) combination of excitatory and inhibitory units. In the given form illustrated in Fig 1A, the matrix 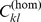 is strictly speaking not homogeneous but contains an asymmetry. This, however, did allow for mapping our model to the Kuramoto network by adapting an approach of Frank and co-workers [31]. In essence, we applied a change in variables yielding a fully homogeneous coupling matrix. For the sake of legibility we set 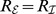, 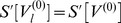 and 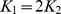. Then 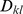 simplified to 
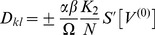
 where the ‘
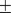
’-sign discriminates rows that correspond to the excitatory/inhibitory neural masses 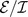, respectively. By this simplification the key feature of the system (9), the asymmetry in the coupling matrix, remained untouched.

We further transformed the system into a rotating frame at frequency 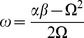 by defining [65], [66]

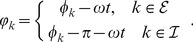
(17)


After abbreviating the constants 
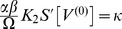
, the phase dynamics (9) became 
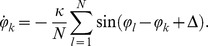



In line with Kuramoto's analysis we introduced a mean (or cluster) phase 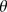 and amplitude 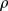 in terms of 
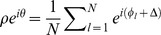
 that represent well-known order parameter(s). Finally, substituting


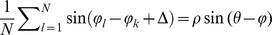
 yielded



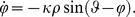



Here we dropped the subscript 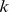 because the equation applies for all nodes. This dynamics agrees entirely with the Kuramoto model, apart from the fact that we here considered 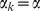 and 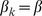 by which all oscillators' natural frequencies 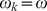 agree. Due to this correspondence to the Kuramoto model, we could conclude that the system with equal delays 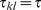 and ‘homogeneous’ coupling matrix 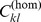 can generate synchronized states provided the overall coupling strength 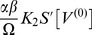 is properly chosen.

### Numerical assessments

Both distributed delays 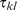 and heterogeneous coupling called for numerical assessments, particularly when it came to the stability of solutions. We performed numerical simulations of the coupled neural masses (1) using a conventional Euler-forward integration scheme with time step 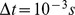 over a time span of 10 s. We verified the appropriateness of this simple implementation against a more elaborate predictor/corrector integrator [67], which revealed little to no difference but demanded far more numerical resources. The simulated network consisted of 500 nodes (250 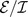 pairs) with 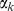 and 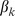 being randomly drawn from uniform distributions (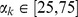 and 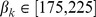) to mimic distributed natural frequencies per 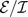-pair of nodes. Although randomly drawn, these sets were fixed across simulations trials. Initial conditions where chosen randomly and did vary between trials; 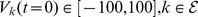, 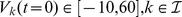 and similarly for 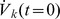. The external input 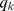 was set to 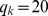, 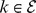, 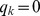, 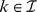. Coupling between masses was achieved by using the sigmoidal activation function 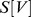 that was given as 
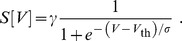



The fraction in this equation may be interpreted as the cumulative distribution function of the normal distribution 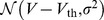 of the firing thresholds 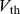 across the population, whereas the constant 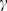 is just a scaling factor [68]. In the simulations we used the following values for the activation function parameters: 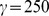, 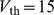 and 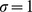.

In order to compare the numerics with our analytical results, we determined the phase values 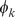 from the simulated potentials 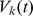 by means of the Hilbert phase. To this end, we first determined oscillation frequency 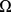 as the lowest frequency with a coinciding peak in the power spectra for all nodes. Voltage traces 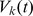 were band-pass filtered using a 1-st order bi-directional Butterworth filter in the frequency band 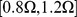. For each sample in the interval 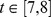, phase values 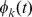 were then calculated as the angle of the analytical signal. By restricting analysis to that interval we avoided transient behavior as well as possible filter artifacts. The so-determined 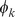 contained the frequency component 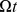, which we first subtracted to obtain 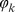. Then, we opted to compute phase distributions over the time interval 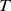 and over successive trials. The (circular) mean phase, however, differed from trial to trial because of the randomly chosen initial conditions (see above). Hence for every trial we shifted phases by the mean phase of the excitatory population prior to concatenating trials. By this, the phase distributions of the excitatory phases always became centered around zero and the inhibitory phases were considered relative values. The mean phase per trial was given as 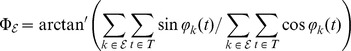
where 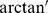 denotes the quadrant-corrected inverse tangents.

The distributions displayed in the figures are the phase distributions obtained from 100 simulated trials with different initial conditions. As said, parameter values were identical across trials. For the simulations involving 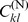 connectivity, we used a binary form of the 66 areas parcelated Hagmann et al. matrix [24], [32] as 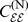 block; see Fig 7 and 8A for the coupling scheme and 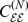, respectively. Functional connectivity was quantified as phase coherence given by 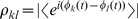. We performed one hundred simulation runs of 10 s for each 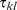 distribution with different initial conditions and 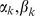 and averaged the 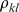 matrices over these runs. This was done to avoid high 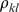 values due to common oscillation frequency alone. For each run, data in the interval 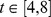 was used to determine 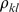. The overall coupling strengths were set to 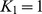 and 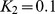 for the 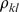 matrices displayed in Fig 8. Structure-function correspondence 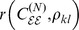 was quantified as the Pearson correlation coefficient between the lower triangular parts of both matrices to avoid spurious correlation values due to common terms along the diagonal.

### Eigenvalues of the linear system

To estimate the oscillatory regime of the system of coupled Freeman neural masses (1) we considered the linearized dynamics (8) for 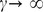 which in general reads 
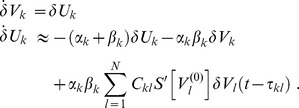



Assuming all delays 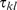 to be (very) small we expanded 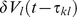 in the sense of Taylor and approximated up to the linear order in 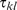: 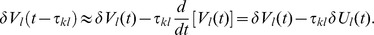



For the sake of legibility we here considered a single isolated 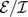-pair with 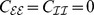 and 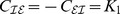. We also assumed equal delays, i.e. 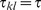. Then, we found the resulting linear dynamics as 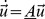 where we abbreviated 
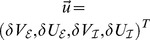
 and 
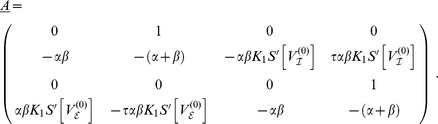



The matrix 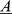 came with eigenvalues 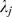


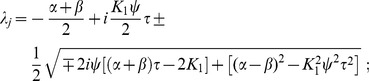
where we abbreviated 
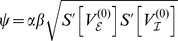
. These eigenvalues had the following real and imaginary parts 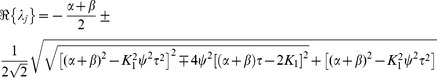


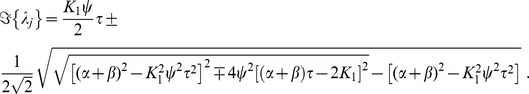



A necessary condition for the existence of a stable limit cycle, and hence for the system (1) to display oscillatory behavior, is that the fixed point 
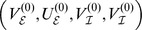
 is unstable. This means that for at least one of the conjugate pairs 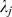, 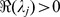 must hold, which was indeed the case irrespective of 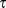. The corresponding 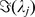 then provided a rough estimate for the frequency 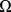 as a function of 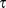, as shown in Fig 2 (solid line). In the particular case of 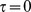 we found 
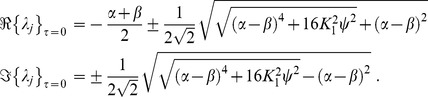
revealing that for 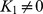 the imaginary parts did not vanish, i.e. the 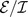-unit always displayed oscillations around the fixed point 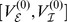 because the sigmoid's derivative is positive definite: 
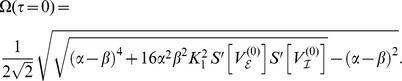



Further, for the real-part to be positive the coupling constant 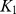 had to be sufficiently large and the intrinsic damping 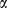 and/or 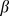 sufficiently small but finite, because of 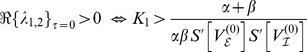
where we note that 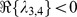.

### Distinct time scales

For the separation of time scales underlying all the major approximations in the current study we considered the case in which two distinct time scales are present in the system of coupled neural masses: the oscillation described by the (mean) frequency 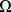 and its corresponding period 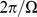 — from here-on referred to as fast time scale — as well as a slower time scale, on which the dynamics of 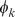 evolve. In what follows we will verify the expression for 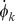 in (9) and show how the separation of time scales enabled us to determine the role of 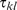 in the convolution 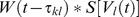. As is conventional in multiple-scaling approaches, we set the time 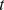 as ‘fast’ time and the ‘slow’ time as 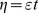 with 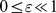. For the sake of legibility we here adopted the dot-notation for temporal derivatives 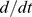 and further abbreviated partial derivatives as 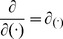.

We denoted the deviation of the voltage from its fixed-point as 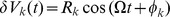 where we assumed that 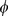 evolved on the slow time 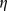, i.e. 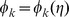. Note that an equivalent approach can be adopted for the amplitude dynamics, i.e. 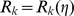, which is referred to as the *slowly varying amplitude approximation*. As we were primarily interested in the 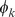 dynamics, we regarded amplitude 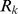 as constant on both time scales; see *Discussion* section. By this we could readily apply the chain rule and obtained 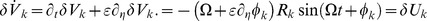
where the last equality follows from (8). For the derivative 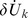 we found 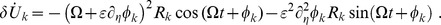



Next we considered the expression (9) for 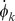, where we emphasize that 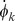 could here be identified as 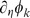, since 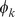 evolves only on the slow time scale. To anticipate: (9) discarded all terms evolving on the fast time scale, i.e. all 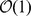 terms in favor of 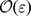 terms and higher. To show this, recall that the right-hand side of (9) read 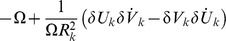


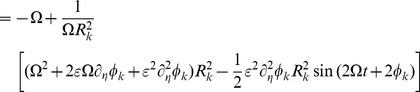


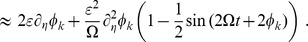



In words, only expressions evolving on the slow time scale were retained, i.e. only 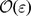 and 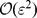-order terms. When focusing on the slow time sale 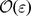 and discarding the even slower time scale 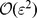, we could conclude that, up to a constant, 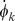 is given by (9); note that we here applied the so-called *two-timing* method; see, e.g., [23], [69], [70].

As said, we used the constancy of 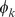 (on the fast time scale 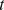) to evaluate the convolution term 
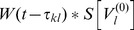
. We exploited the description in two time scales to justify the transformation of the delay 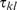 into phase shifts 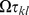. We explicitly evaluated the integral 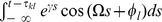 to show that (10) is its 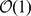 result. For the sake of readability we dropped the explicit time dependence of 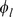 whenever possible.

First, by integrating by parts twice we obtained 
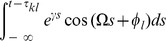


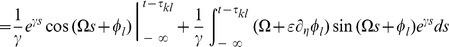


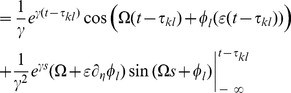


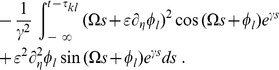



Then, when discarding 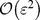 terms, we found that the integral with which we started appeared again on the right-hand side. This allowed us to write 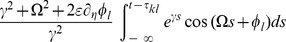


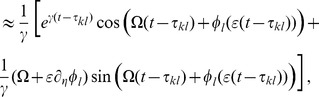
which resulted in 
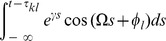


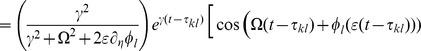


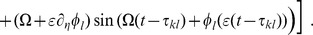



With this form we could finally express the convolution terms as 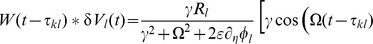


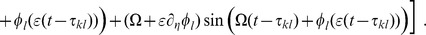



For 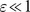 time scales are sufficiently separated to ignore all 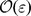 terms by which we arrived at (10).

The derivation of (10) required the intuitive assumption 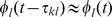, which might be motivated by a (relatively) small-delay approximation. Consider the Taylor expansion of 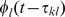 around 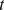, which reads 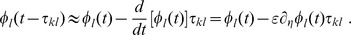



If 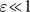 and 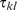 is of the order 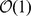, i.e. of the same order of magnitude as the oscillatory period 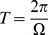, then one may conclude that the approximation 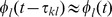 is valid. Note that this consistent with [71]. In particular, when 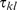 is of order 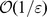, i.e. of the same order of magnitude as the slow time scale, the approximation 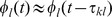 fails.
